# Extraction of tobacco extract from discarded tobacco leaves for cigarette yarns dyeing and neophytadiene separation

**DOI:** 10.3389/fchem.2025.1682505

**Published:** 2025-11-21

**Authors:** Long Wang, Weihua Chen, Yuqi Wan, Aimin He, Jiayin Liu, Xuemin Hu

**Affiliations:** 1 China Tobacco Hebei Industrial Co., Ltd., Shijiazhuang, China; 2 College of Textile and Garments, Hebei University of Science and Technology, Shijiazhuang, China

**Keywords:** discarded tobacco leaf, tobacco absolute oil, tobacco extract, neophytadiene, cigarette yarns dyeing

## Abstract

A continuous process integrating extracting valuable compounds from waste tobacco leaves for cigarette yarns dyeing and neophytadiene separation was developed, enabling sequential production of tobacco crude extract, colored yarns, tobacco-derived absolute, and neophytadiene. Systematic comparison of residues from four regions revealed that Lanxiong-derived biomass demonstrated the highest extract yield (25%), providing optimal raw material for scaled neophytadiene production. GC-MS and NMR analyses confirmed >98% purity and structural integrity of purified neophytadiene. When the crude extract was applied directly to cotton yarns dyeing, the dyed yarn with 1.5 wt% tobacco crude extract solution from Zunyi achieved a K/S value of 2.013 and exhibited a yellow-brown hue shift that met visual identification requirements for cigarette yarns. The dyed yarns retained over 90% tensile strength of untreated controls, while elongation and unevenness indices showed no statistically significant alterations. This integrated approach establishes a green and sustainable technological route for valorizing tobacco waste within circular economy frameworks.

## 1 Introduction

Tobacco, as one of the globally significant cash crops, generates substantial biomass residues during cultivation and processing (Zeng et al., 2023; Zhou et al., 2020), including stalks (Cui et al., 2022), midribs, and non-compliant leaves (Musharraf et al., 2012), necessitating advanced valorization strategies (Yuan et al., 2022). The disposal of these waste streams not only exerts environmental pressures but also represents a loss of potentially valuable resources (Liu et al., 2024; Yang et al., 2022). Integrated biorefining of such waste streams represents a critical pathway toward sustainable resource circularity (Wang et al., 2015). With the growing emphasis on sustainable development, the strategic valorization of these byproducts has emerged as a critical research focus for enabling green transformation within the tobacco industry (Araújo and Costa, 2019; Bai et al., 2025).

**FIGURE 1:**
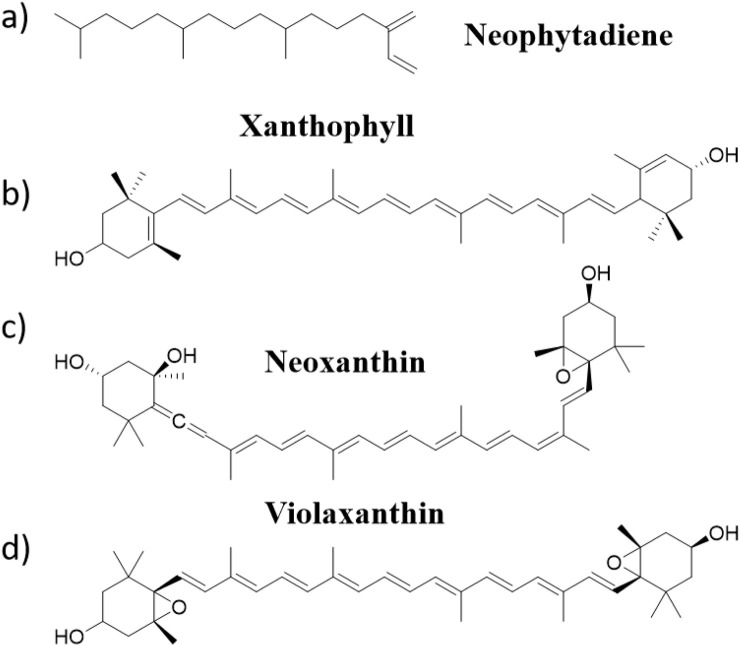
The structural formula of **(a)** Neophytadiene, **(b)** XanthophyⅡ, **(c)** Neoxantin, **(d)** Violaxanthin.

Tobacco leaves are known to contain over 4,000 structurally diverse phytochemicals exhibiting significant bioactivity at minimal dosages (Nielsen et al., 2014; Li et al., 2023). Among these, neophytadiene (Figure 1a) functions as a key aroma-transport mediator, facilitating the transfer of volatile flavor compounds into combustion aerosols (Eldemerdash et al., 2022; Li et al., 2020) Tobacco absolute as a high-purity distillate possesses superior olfactory characteristics, exceptional compatibility with flavor matrices, and efficacy in augmenting tobacco-specific sensory profiles by amplifying foundational notes while suppressing off-odors. Strategic recovery of neophytadiene from tobacco absolute enables dual valorization: enhancing organoleptic quality in tobacco products and creating high-value biomass-derived materials for sustainable coloration (Oyagbemi et al., 2019). Current extraction of tobacco crude extracts and purification of neophytadiene primarily rely on conventional toxic organic solvent extraction and thermal distillation techniques (Shomron et al., 2022; Gonzalez et al., 2022). However, these methods are associated with substantial energy consumption (Lin, 2019), elevated costs, and environmental concerns, necessitating the development of green and efficient alternatives (Jing et al., 2022; Kumar Pandey et al., 2022).

While tobacco provides significant economic benefits, it also poses serious health risks to the public (Hirn et al., 2020). Tar reduction and harm mitigation are particularly effective. However, low-tar cigarettes often have poor taste and comfort, making them less acceptable to consumers. Tobacco fragrances have the roles of enriching tobacco aroma, improving inhalation quality, and enhancing the quality of tobacco leaves. They are important materials for improving the physicochemical properties and fundamental taste of products and have become indispensable additives in tobacco products, compensating for the shortcomings of tar reduction and harm mitigation (Rodu and Godshall, 2006). The fragrances also have a positive effect on people’s emotions and behaviors, helping to reduce anxiety, improve mood, and stimulate creative thinking (Lenochová et al., 2012). In cigarette manufacturing, flavorant-impregnated yarns are commonly integrated into filter rods to achieve the effect of enhancing aroma (Supplementary Figure S1). As the demand for cigarette filters yarns grows, the quality of yarns products is also becoming important (Churchill and Behan, 2010). To emphasize distinctive product attributes, these yarns are typically colored using toxic synthetic dyes, which may pose potential threats to human health. Therefore, the utilization of pigments derived from waste tobacco leaves for dyeing yarns could be of considerable significance. Mature tobacco contains various carotenoids, including lutein, neoxanthin, and violaxanthin (Figures 1b–d), which could be used as dyes for dyeing cellulose fibers (William and Johng, 1982). Yilmaz F et al. investigated the usability of tobacco industrial waste as natural dye sources and antibacterial agents for cotton fabrics (Yilmaz and Bahtiyari, 2020). However, the application of tobacco extract for dyeing cigarettes yarns and its subsequent recycling for the isolation of neophytadiene has been scarcely studied.

Herein, an eco-friendly extraction protocol for recovering tobacco crude extracts from waste leaves was designed. The intermediate products obtained during neophytadiene purification were subsequently employed for yarns dyeing. Through process optimization, neophytadiene purity was enhanced and the chromatic properties and mechanical integrity of the dyed yarns were evaluated. These findings demonstrate a novel approach for the sustainable valorization of tobacco processing byproducts.

## 2 Experiment

### 2.1 Materials

The aforementioned waste tobacco leaves were sourced from four geographically distinct regions: Longyan, Lanxiong, Zunyi, and Yongzhou, which are named A, B, C, and D (Most tobacco raw materials required for the experiment were sourced from southern regions. The four production areas—Longyan, Lanxiong, Zunyi, and Yongzhou—were geographically dispersed across provinces with distinct environmental conditions and substantial temperature variations. This spatial and climatic heterogeneity enabled these regions to serve as comparative controls, facilitating differentiation of tobacco raw materials to a certain extent). All leaves were harvested at physiological maturity and stored under shaded, arid conditions for a minimum of 7 days prior to use. Cotton yarns, 32s. Cyclohexane (≥99.9%) and phosphomolybdic acid were procured from Aladdin Biochemical Technology Co., Ltd. (China). Aluminum potassium sulfate dodecahydrate, and anhydrous ethanol (analytical grade) were purchased from Tianjin Yongda Chemical Reagent Co., Ltd. All the reagents were used without further purification.

### 2.2 Extraction of tobacco extract

Dried waste tobacco leaves were pulverized and sieved. The resultant powder was mixed with 80% anhydrous ethanol at a liquid-to-solid ratio of 3:1 (v/w). The mixture was refluxed at 80 °C for 3 h in an oil bath, followed by vacuum filtration. The filtrate was concentrated to complete ethanol removal using rotary evaporation to obtain crude tobacco extract, with yield calculated.

### 2.3 Dyeing process for cotton yarns with tobacco extract

Before tobacco extract was applied to cotton yarns, mordant dyeing were carried out for 30 min with aluminum potassium sulfate dodecahydrate as mordant (3 wt% o.w.f). Then, the tobacco extract was dissolved in water with the content 0.5 wt%, 1.0 wt% and 1.5 wt%. Subsequently, the cotton yarns were immersed in dye solution at 40 °C. The temperature was increased to 85 °C with heating rate of 1 °C/min and kept for 60 min. After cooling to the room temperature, the cotton yarns were removed, washed with water and soap respectively. The soaping process parameters were as followed. The bath ratio was 60:1, and the concentration of electro-neutral soaping solution was set 2 g/L. The cotton yarns were soaped at 65 °C for 15 min, and then washed with water and dried.

### 2.4 Preparation of tobacco absolute

Tobacco extract (10 g) or post-dyeing recovered extract was dissolved in anhydrous ethanol (50 mL) within a 250 mL conical flask. After 1 h heating at 60 °C in a water bath, hot filtration was performed. The filtrate was concentrated under reduced pressure at 45 °C until no liquid droplets formed (20 min duration), yielding tobacco absolute.

### 2.5 Purification of neophytadiene

Neophytadiene was purified by column chromatography, with cyclohexane as the eluent and phosphomolybdic acid as the developing agent.

### 2.6 Characterization

Scanning electron microscopy (SEM, S-4800, Hitachi, Japan) was used for the morphological analysis of cotton yarns. Absorbance measurements were performed with a Visible Spectrophotometer from Shanghai Yuan Analytical Instrument Co., Ltd. The infrared spectra of extract samples from various locations were determined using a Bruker VERTEX 70 Fourier Transform Infrared (FTIR) Spectrophotometer. Color parameters (L^*^, a^*^, b^*^, C^*^, h^*^, ΔE^*^, K/S (Equation 1)) of dyed yarns were measured using a computerized colorimeter (Color i5, Datacolor, American). The absolute oil and neophytadiene in tobacco were analyzed using a GC-MS (Shimadzu GCMS-QP2020, Japan) equipped with an DB-5 MS column (30 m * 0.25 mm * 0.25 µm). The injector temperature was set at 250 °C, with a helium carrier gas flow rate of 40 mL/min. The temperature program was initiated at 60 °C, ramped up at 20 °C/min to 260 °C, and held at 260 °C for 11 min. Tensile properties including breaking force (F), elongation (L), yarn irregularity (D), elongation at break (E) were evaluated before and after dyeing via universal material testing machine. Neophytadiene was characterized by ^1^H-NMR and ^13^C-NMR spectra with a Bruker 400 AVANCE instrument (400 MHz and 126 MHz) at room temperature.

### 2.7 ANOVA data analysis

Statistical analysis for color difference (ΔE^*^) and breaking force (F) was conducted using the SPSSAU project (version 25.0 [Online Application Software]. Retrieved from https://www.spssau.com.), and the IBM SPSS Statistics (version 29.0.2.0 Build 20). A two-way ANOVA was specifically employed to assess the interactive effects of dye source and concentration on yarns performance metrics.

## 3 Results and discussion

### 3.1 Sustainable tobacco extract from discarded tobacco leaves

**FIGURE 2:**
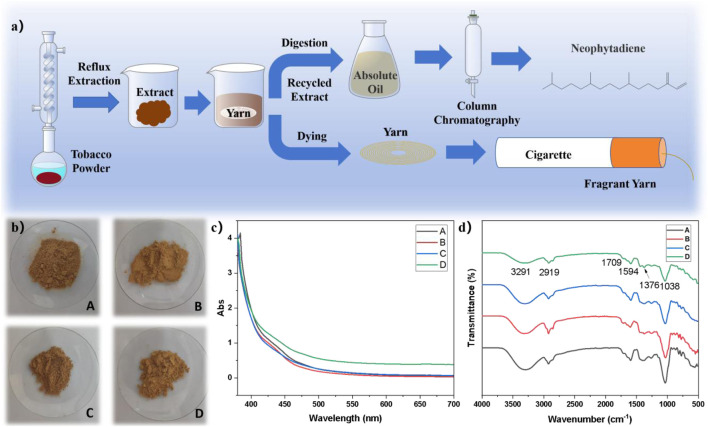
**(a)** Schematic of the neophytadiene purification process and yarn dyeing procedure; **(b)** Images of tobacco waste powder samples from different origins: Longyan (A), Lanxiong (B), Zunyi (C), and Yongzhou (D); **(c)** Absorbance spectrum of the tobacco extract; **(d)** FTIR spectrum of the tobacco extract.

Figure 2a illustrated the integrated process for neophytadiene purification and yarns dyeing utilizing waste tobacco leaves. A crude extract was obained using a composite solvent extraction from tobacco powder. The crude extract was then diluted to create a dye solution for coloring cotton yarns. After dyeing, the extract was recovered for the extraction of neophytadiene, thereby achieving multiple utilizations of waste tobacco. Regionally sourced tobacco powders depicted in Figure 2b were subjected to reflux extraction with 80% ethanol at 100 °C for 3 h followed by vacuum filtration. The resulting filtrate underwent complete ethanol removal via rotary evaporation at 45 °C to afford tobacco crude extract (Supplementary Figure S2). Absorbance spectra of extracts from different geographical origins were compared in Figure 2c. FTIR analysis in Figure 2d identified a characteristic absorption peak at 3,291 cm^−1^ was attributable to O-H stretching vibrations. The peak at 2,919 cm^−1^ and 1,465 cm^−1^ corresponded to C-H bond stretching and bending vibration (Afzal et al., 2018). The peak at 1,709 cm^−1^ corresponded to the C=O bond stretching vibration of ketones or esters and the C-O stretching vibration was indicated at 1,031 cm^−1^ (Li et al., 2024). The FT-IR results confirmed that the extract contained alkanes, alcohols, phenols, aldehydes, ketone, esters, and aromatic compounds. Cotton yarns were subjected to exhaust dyeing in crude extract-based liquor for 1 h employing a pre-mordanting protocol. Subsequent soaping and drying procedures produced dyed yarns for cigarette. The recycled crude extract after dying was dissolved in anhydrous ethanol under heating at 60 °C for 1 h with subsequent hot filtration. The filtrate was further concentrated under reduced pressure at 45 °C until cessation of liquid droplet formation to yield tobacco absolute. Final isolation of neophytadiene was accomplished through column chromatography. This approach demonstrated significant potential for valorizing waste-derived resources while establishing circular economy pathways in tobacco processing industries.

**FIGURE 3:**
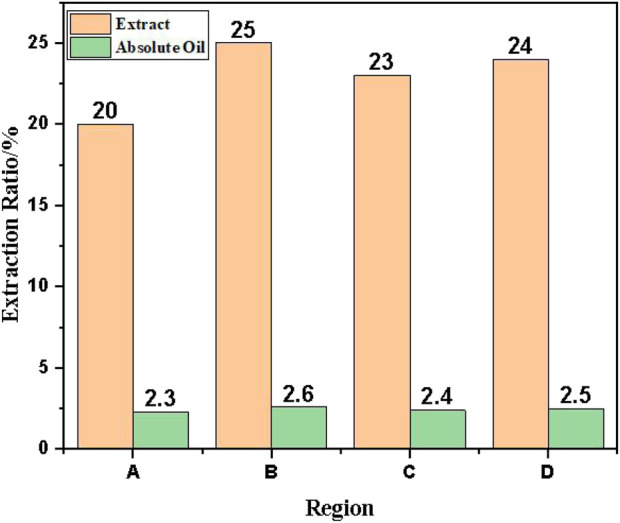
Extraction yields of tobacco crude extracts and absolute oils from four production regions (Longyan (A), Lanxiong (B), Zunyi (C), Yongzhou (D)).

Extraction yields of tobacco crude extracts and absolute derived from waste leaves originating in Longyan (A), Lanxiong (B), Zunyi (C), and Yongzhou (D) were presented in Figure 3. Extraction yield served as a critical metric for evaluating process efficiency, with significant implications for optimizing operational parameters and enhancing resource utilization. Pronounced regional variations in extract yields were observed, with Lanxiong (B) exhibiting the highest yield, followed by Zunyi (C) and Yongzhou (D), while Longyan (A) demonstrated the lowest values. Across all regions, the absolute extraction yield was consistently lower than the crude extract yield, indicating a hydrophobic fraction. This variability may be attributable to differences in the content of extractable compounds in Lanxiong-derived biomass or the superior extraction efficiency under the applied process conditions. Regional differences could potentially be associated with variations in cultivar characteristics, maturity stage, harvest timing, and post-harvest processing protocols (Hong et al., 2022).

### 3.2 Morphology and properties of dyed yarns

**FIGURE 4:**
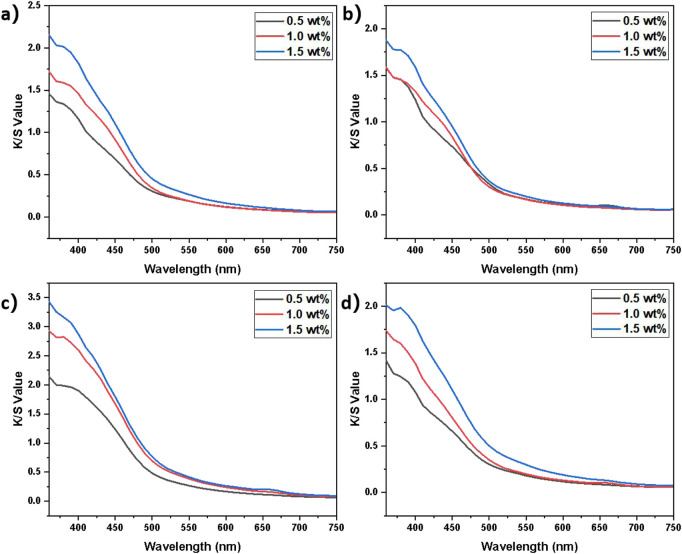
K/S values of yarns dyed with tobacco extracts at varying concentrations, sourced from **(a)** Longyan, **(b)** Lanxiong, **(c)** Zunyi, and **(d)** Yongzhou.

Figure 4 presented the K/S profiles of yarns dyed with tobacco crude extracts from Longyan (A), Lanxiong (B), Zunyi (C), and Yongzhou (D) at varying concentrations of tobacco extracts, showing characteristic absorption maxima at 440 nm. K/S values serve as a crucial indicator of dye adsorption capacity onto fibers and are intrinsically correlated with dye concentration, substrate properties, and dyeing conditions. A significant increase in K/S values was observed as the extract concentration increased, indicating enhanced color depth at higher dye loads within the operational threshold. Specifically, the dyed yarns with 1.5 wt% extract concentration from Zunyi-derived exhibited the highest K/S value (2.013), demonstrating superior dyeing performance. Comparatively, the Yongzhou-derived extract showed lower K/S values under identical conditions, which may be attributed to differences in bioactive component content or structural arrangement. Similar K/S profile shapes were observed across regions, but differing curve slopes were recorded, potentially associated with variations in chromophore molecular size, morphological state, and polar characteristics. Notably, despite fluctuations in K/S values, a consistent profile trend was maintained across concentration intervals, indicating reproducible and stable dyeing behavior of the tobacco extracts within the tested concentration range. This finding holds significant implications for optimizing extraction and dyeing protocols, thereby enhancing process efficiency and dyeing quality in practical applications. Collectively, the differential dyeing performance of region-specific extracts established in Figure 4 laid the groundwork for developing ecologically sustainable textile dyeing technologies.

Colorimetric parameters of yarns dyed with tobacco crude extracts at various concentrations were summarized in Table 1, including Luminance (L^*^), Green-red coordinate (a^*^), Blue-yellow coordinate (b^*^), Chroma (C^*^), Hue angle (h^*^), Color difference (ΔE^*^), and Color depth (K/S). These indicators provided a critical assessment of coloration efficacy and chromatic stability. The L^*^ value ranged from 0 to 100, representing a transition from black to white. A decreasing trend in luminance (L^*^) values was observed with increasing extract concentration, indicating increased color depth and decreased lightness at higher dye loads. For example, in Group A, the L value decreased from 78.46 at 0.5 wt% to 75.07 at 1.5 wt%.

**TABLE 1:** The color properties of the dyed yarns.

Name	Luminance (L^*^)	Green-red coordinate (a^*^)	Blue-yellow coordinate (b^*^)	Chroma (C^*^)	Hue angle (h^*^)	Color difference (DE^*^)	​
A (0.5 wt%)	78.46	1.67	22.95	23.01	85.85	24.68	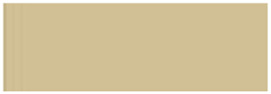
A (1.0 wt%)	78.11	1.54	28.46	28.50	86.91	29.81	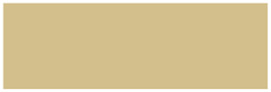
A (1.5 wt%)	75.07	2.60	28.04	28.16	84.70	30.82	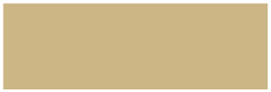
B (0.5 wt%)	78.88	1.07	25.33	25.35	87.58	26.60	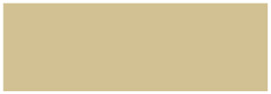
B (1.0 wt%)	79.26	0.71	28.20	28.21	88.55	29.11	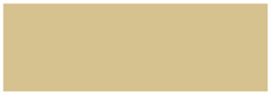
B (1.5 wt%)	77.64	1.32	28.68	28.71	87.36	30.18	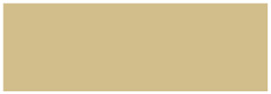
C (0.5 wt%)	74.85	2.58	30.26	30.36	85.13	32.87	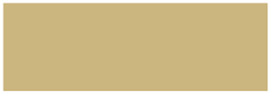
C (1.0 wt%)	70.89	3.85	31.35	31.59	82.99	35.88	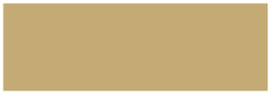
C (1.5 wt%)	69.9	3.68	31.61	31.83	83.35	36.61	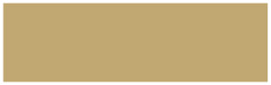
D (0.5 wt%)	78.76	1.84	22.61	22.68	85.34	24.25	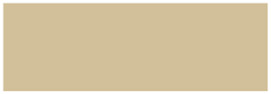
D (1.0 wt%)	77.74	1.34	25.40	25.44	86.99	27.17	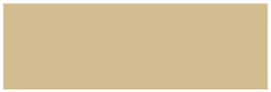
D (1.5 wt%)	73.92	3.02	26.47	26.64	83.50	30.07	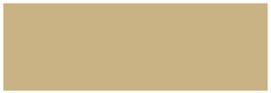

Fluctuations in the green-red (a^*^) and blue-yellow (b^*^) coordinates reflected changes in the hue of the dyed yarns. The a^*^ value range was −128 to +128. Within this range, −128a^*^ represented green, gradually transitioning to magenta as it approached +128a^*^; the b^*^ value range was also −128 to +128, where −128b^*^ was blue, gradually transitioning to yellow as it approached +128b^*^. An increasing trend in both a^*^ and b^*^ values was observed for most samples with increasing concentration, indicating a^*^ color shifted towards reddish-yellow tonalities (Additional black from L^*^ value). For instance, in Group B, as the concentration increased from 0.5 wt% to 1.5 wt%, the a^*^ value increased from 1.07 to 1.32, and the b^*^ value increased from 25.33 to 28.68.

Variations in chroma (C^*^) and hue angle (h^*^) reflect chromatic intensity and directional hue shifted in dyed yarns. Concentration-dependent increases in both C^*^ and h^*^ values were observed across most specimens, indicating enhanced color vividness and pronounced hue expression. For instance, in Group C, C^*^ values elevated from 30.36 to 31.83 and h values increased from 85.13 to 83.35 as concentration escalated from 0.5 wt% to 1.5 wt%. Color difference (ΔE^*^), serving as a quantitative indicator of chromatic deviation between dyed and undyed yarns, demonstrated positive correlations with concentration increments. Specifically, ΔE^*^ values for Group D rose from 24.25 at 0.5 wt% to 30.07 at 1.5 wt%. The color difference was then analyzed by two-way ANOVA using SPSSAU with two sets of additional ΔE^*^ values (Supplementary Table S1), and the results were presented in Table 2. As revealed by variance analysis, both origin (A) and concentration (B) factors exerted highly significant independent effects on color difference (ΔE^*^), with F-values of 451.888 (p < 0.001) and 238.692 (p < 0.001), respectively. The interaction between origin (A) and concentration (B) was statistically significant (F = 6.287, p < 0.001). The results indicated that the effect of one factor (e.g., concentration) on the color difference dependsed on the level of the other factor (e.g., origin) and variations in raw material sources and dye concentration independently contributed to dyeing effect of the yarns.

**TABLE 2:** Two-way ANOVA for color difference.

Source of variation	Sum of squares (SS)	*df*	Mean square (MS)	*F*	*p*-value
Intercept	31803.372	1	31803.372	138924.854	0.000**
Origin	310.345	3	103.448	451.888	0.000**
Concentration	109.285	2	54.643	238.692	0.000**
Source region*concentration	8.635	6	1.439	6.287	0.000**
Residual	5.494	24	0.229	​	​

*R* ^2^ = 0.987.

*p < 0.05 and **p < 0.01.

Tunable coloration of yarns was achieved through the strategic application of tobacco crude extracts derived from waste leaves. Chromatic attributes-including luminance, hue, chroma, and staining fastness—were effectively controlled through concentration modulation of the extracts, establishing viable pathways for value-added valorization of tobacco processing residues.

**FIGURE 5:**
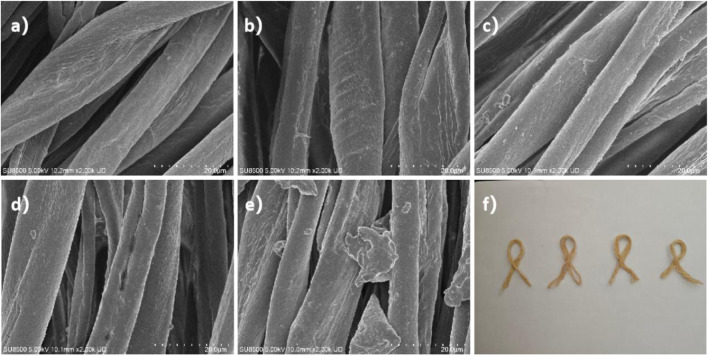
Surface morphology of **(a)** untreated control yarn; Dyed yarn morphologies with tobacco crude extracts sourced from **(b)** Longyan, **(c)** Lanxiong, **(d)** Zunyi, and **(e)** Yongzhou; **(f)** Macroscopic appearance of dyed yarns of four regions.

Figure 5 characterized macro- and micro-morphological alterations in yarns before and after tobacco extract dyeing. Figure 5a revealed a clean surface of undyed control yarns, with fibers exhibiting characteristic band-like bundled arrangements. Distinct surface grooves (1–2 μm width) were observed without particulate deposits, indicating effective removal of sizing agents and impurities during pretreatment. As demonstrated in Figures 5b,c,e,f, yarns dyed with tobacco extracts from different origins displayed continuous and dense particulate layers uniformly covering fiber surfaces. No discernible cracks or delamination were detected, confirming the formation of homogeneous and stable physically adsorbed layers of tobacco pigments on yarn substrates.

As illustrated in Figure 5b, the Longyan sample exhibited the highest particle packing density with localized slight agglomeration. Particles in the Lanxiong sample (Figure 5c) demonstrated relatively uniform dimensions and the lowest surface roughness, indicating superior dispersion stability of the extract from this origin. Microcracks were observed in the Zunyi sample (Figure 5d), though they do not compromise overall coverage. Particles in the Yongzhou sample (Figure 5e) displayed oriented alignment, potentially attributable to capillary adsorption within microgrooves along the fiber axis.

The yarns shown in Figure 5f presented a homogeneous tan coloration after dyeing, free from staining or ring dyeing phenomena, essentially meeting the uniformity requirements for textile dyeing. Consequently, the tobacco extract formed a continuous coloration layer on the cotton yarn surface. While subtle variations in particle morphology and distribution existed among extracts from different geographical origins, their impact on macroscopic color uniformity remained negligible. These morphological observations provided a foundation for subsequent optimization of natural dye formulations through origin selection and enhancement of color fastness.

The dyed yarn was subsequently immersed in peppermint essence, and the gas chromatography analysis of the peppermint essence is shown in Supplementary Figure S3. The fragrant yarn was then wrapped around a filter stick, and its fragrance value was measured to be 81 (Supplementary Figure S4).

Table 3 presented the tensile performance data of yarns before and after dyeing, including breaking force (F), elongation (L), yarn irregularity (D), and elongation at break (E). These parameters were essential for evaluating the impact of tobacco extract dyeing on yarn mechanical properties. Regarding breaking force (F), dyed yarns generally exhibit lower values compared to undyed controls with the retention rate more than 90%. For instance, Group A demonstrated a breaking force of 387.2 cN after 0.5 wt % dyeing, representing a reduction from the control value of 401.9 cN. This decrease was presumably attributable to interactions between chemical constituents in the tobacco extract and yarns, potentially compromising inter-fiber bonding.

**TABLE 3:** Yarn tensile strength before and after dyeing.

Name	F (cN)	L (mm)	D (cN/t)	E (%)
Blank	401.9	31.67	22.08	6.33
A (0.5 wt%)	387.2	24.27	21.27	4.85
A (1.0 wt%)	435.4	35.06	23.91	7.01
A (1.5 wt%)	387	32.22	21.26	6.44
B (0.5 wt%)	343.2	29.83	18.85	5.96
B (1.0 wt%)	417.5	30.64	22.93	6.12
B (1.5 wt%)	389.3	33.88	21.38	6.77
C (0.5 wt%)	392.3	27.25	21.55	5.44
C (1.0 wt%)	387.2	29.46	21.27	5.89
C (1.5 wt%)	342.8	24.07	18.83	4.81
D (0.5 wt%)	384.9	30.71	21.14	6.13
D (1.0 wt%)	392.5	30.61	21.56	6.12
D (1.5 wt%)	411.5	29.20	22.60	5.83

Elongation (L), reflecting yarn flexibility, showed no significant alterations post-dyeing according to the tabulated data. This indicated that the impact of tobacco extract dyeing on the pliability of the yarns was negligible. Yarn irregularity (D), an important indicator of yarn uniformity (homogeneity), showed reduced values after dyeing. For example, Group C exhibited a mass variation of 18.83 cN/t after dyeing at 1.5 wt%, which was lower than the control value of 22.08 cN/t. This reduction may stem from differences in hydrophilic properties among fibers during the dyeing process. Elongation at break (E), representing the elastic force of the yarns, generally decreased after dyeing. Group D showed an elongation at break of 5.83% after dyeing at 1.5 wt%, a decrease from the control value of 6.33%. This suggested that tobacco extract dyeing may reduce the elastic force of the yarns, potentially associated with changes in chemical structure during the dyeing process. The breaking force was also analyzed by two-way ANOVA (Table 4). The results indicated that different F (cN) samples showed no significant differences (p > 0.05) in concentration and origin (A). This implied that there was no consistent variability between different F (cN) samples regarding these two factors.

**TABLE 4:** Two-way ANOVA results for breaking force.

Source of variation	Sum of squares (SS)	*df*	Mean square (MS)	*F*	*p*-value
Model	1983294.330	7	283327.761	423.537	<0.001
Origin	1526.500	3	508.833	0.761	0.556
Concentration	2213.167	2	1106.583	1.654	0.268
Error	4013.740	6	668.957	​	​
Total	1987308.070	13	​	​	​

*R* ^2^ = 0.987 (Adjusted *R* ^2^ = 0.996).

### 3.3 Purification and properties of neophytadiene

**FIGURE 6:**
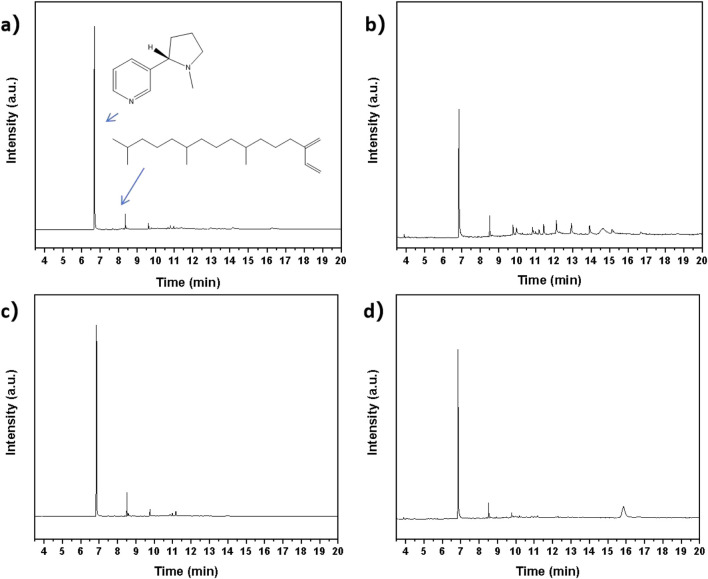
GC/MS chromatograms of tobacco absolute derived from geographically distinct sources: Longyan **(a)**, Lanxiong **(b)**, Zunyi **(c)**, and Yongzhou **(d)**.

Figure 6 presented the Gas Chromatography-Mass Spectrometry (GC/MS) results of absolute oils obtained from extracts originating from four different geographical origins (Longyan (A), Lanxiong (B), Zunyi (C), Yongzhou (D)). Comparative examination of the chromatographic profiles reveled significant presence of nicotine and neophytadiene constituents across all extracts (Endris, et al., 2024; Muzyka et al., 2022). As depicted in Figure 6a, the predominant peak was attributed to nicotine at 7–8 min, while the secondary peak at 8–9 min corresponded to neophytadiene. The distinct presence of nicotine (83.7%, 58.2%, 65.9% and 85.3% for A, B, C and D) in GC/MS spectra confirmed both the stability of alkaloid compounds within the tobacco extract and the efficiency of the extraction protocol. Concurrently, the detection of neophytadiene, a critical nonpolar constituent of tobacco extract (7.7%, 7.8%, 7.5% and 5.1% for A, B, C and D), established a foundation for its subsequent purification and application. Comparative analysis of the chromatographic profiles of all four origins revealed identifiable variations in additional impurity components among the extracts. These compositional differences may be attributed to varying soil conditions, climatic variations, and tobacco cultivars across geographical regions. The GC/MS data demonstrated that the recovered tobacco extract still contained compounds such as nicotine and neophytadiene, which were not fully absorbed by the cotton yarn during the dyeing process. The analysis results provided critical evidence for further research on the chemical composition of tobacco extracts, optimization of extraction techniques, and improvement of dyeing performance.

**FIGURE 7:**
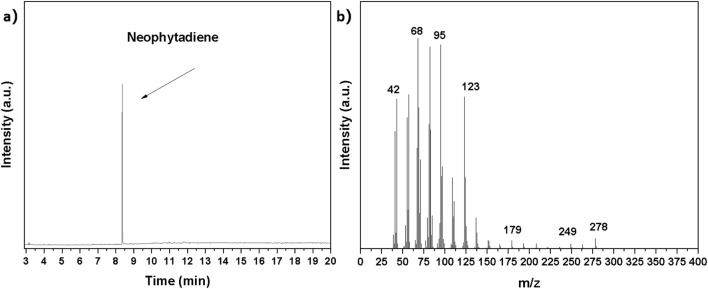
**(a)** Gas chromatography **(b)** Mass spectrometry of purified neophytadiene.

Figure 7 showed the Gas Chromatography-Mass Spectrometry (GC/MS) profile of neophytadiene purified via column chromatography. This chromatogram served as a vital tool for evaluating purification efficacy, directly reflecting the purity and distribution of compounds within the residual components. As observed in Figure 7a, the neophytadiene peak appeared at a retention time of approximately 8 min and was very sharp, indicating high purity. Figure 7b depicted the mass spectrum of this compound, with m/z of 278 corresponding to the molecular ion peak of neophytadiene, and the other fragment peaks also confirmed its structure. The absence of significant extraneous peaks further demonstrated its outstanding purity characteristics. Quantitative analysis confirmed the neophytadiene purity exceeded 98%, demonstrating column chromatography as an effective purification technique for isolating high-purity neophytadiene from tobacco extracts. This methodology represented a promising approach for valorizing waste tobacco leaves and supports future applications of neophytadiene.

**FIGURE 8:**
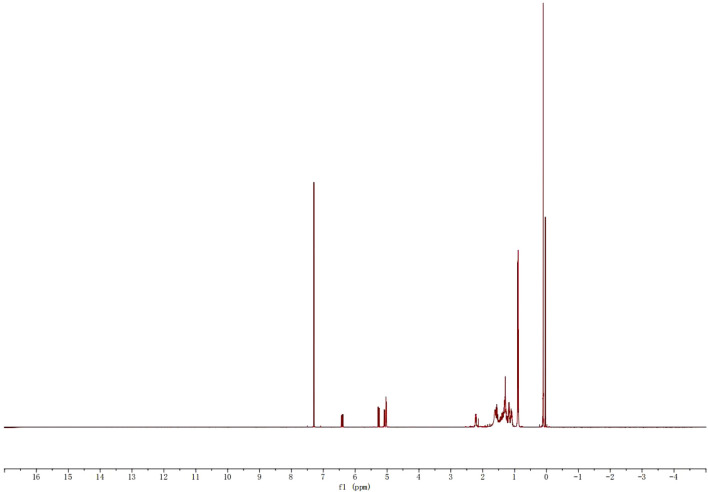
H-NMR spectra of purified neophytadiene.

**FIGURE 9:**
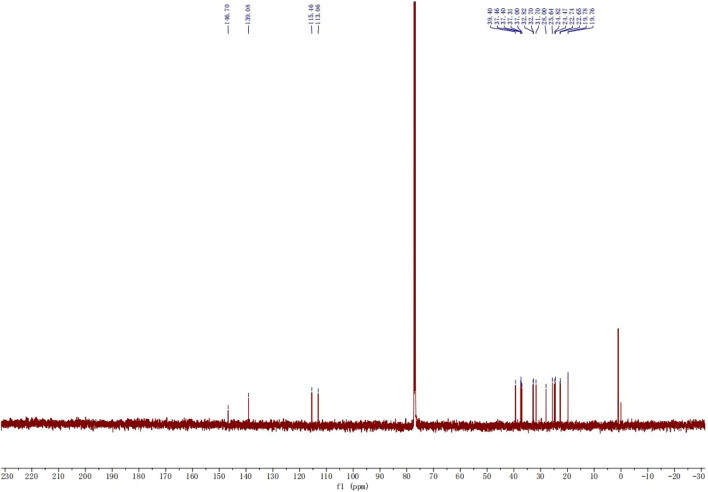
C-NMR spectra of purified neophytadiene.

Figures 8, 9 presented the H-NMR and C-NMR spectra of purified neophytadiene, respectively. In the ^1^H-NMR spectrum (Figure 8), signals at 5–7 ppm were assigned to five protons from two olefinic bonds, while resonances at 0–1 ppm corresponded to twelve protons of four methyl groups. Additionally, the multiplet spanning 1–3 ppm was attributed to nineteen protons from methyl and methylene groups.

^13^C-NMR spectrum exhibited signals at 110–150 ppm corresponding to four olefinic carbons, whereas chemical shifts between 20 and 40 ppm were assigned to sixteen alkyl chain carbons (Bhardwaj et al., 2020). These NMR data unequivocally confirmed the molecular structure of neophytadiene and offered critical spectroscopic validation for further structural elucidation and functional studies. Comprehensive spectral analysis verified both the purity and structural integrity of neophytadiene, providing scientific substantiation for evaluating its potential in extraction, purification, and value-added applications within tobacco-derived extracts.

^13^C NMR (126 MHz, CDCl_3_) δ 146.70, 139.08, 115.46, 113.06, 39.40, 37.46, 37.40, 37.31, 37.00, 32.82, 32.70, 31.70, 28.00, 25.64, 24.82, 24.47, 22.74, 22.65, 19.78, 19.76.

## 4 Conclusion

This study established a green purification protocol for neophytadiene from waste tobacco leaves. Continuous productions of tobacco extract, dyed yarns, absolute oil, and neophytadiene were successfully achieved. Results indicated that the Lanxiong-origin extract yielded the highest extraction efficiency (7.8%), providing optimal feedstock for scalable neophytadiene production. Digestion coupled with column chromatography separation effectively eliminated impurities, with GC-MS and NMR spectra confirming structural integrity and high purity (>98%) of neophytadiene. Coloration experiments revealed concentration-dependent enhancement of K/S values at 440 nm, with the Zunyi 1.5 wt% tobacco crude extract solution dyed sample achieving a K/S value of 2.013. The hue shift toward yellowish-brown satisfied visual identification requirements for cigarette yarns. The dyed yarns retained >90% tensile strength relative to undyed controls, with no significant alterations in elongation or mass variation.

## Data Availability

The original contributions presented in the study are included in the article/Supplementary Material, further inquiries can be directed to the corresponding authors.
