# Supplementary material for: Extraction of tobacco extract from discarded tobacco leaves for cigarette yarns dyeing and neophytadiene separation
Source: Front Chem. 2025 Nov 21;13:1682505. doi: 10.3389/fchem.2025.1682505 (PMC12679387; doi:10.3389/fchem.2025.1682505)
Supplement: Supplementary file 1 [file DataSheet1.docx]

Supplementary Material

Extraction of Tobacco Extract from Discarded Tobacco Leaves for Cigarette Yarns Dyeing and Neophytadiene Separation

Long Wang^1^, Weihua Chen^1*^, Yuqi Wan^2^, Aimin He^1^, Jiayin Liu^2^, Xuemin Hu^2*^

^1^China Tobacco Hebei Industrial Co., Ltd., China

^2^College of Textile and Garments, Hebei University of Science and Technology, Shijiazhuang,

*** Correspondence:**

Weihua Chen, chenweihua1622@163.com

Xuemin Hu, [huxuemin@hebust.edu.cn](mailto:huxuemin@hebust.edu.cn)


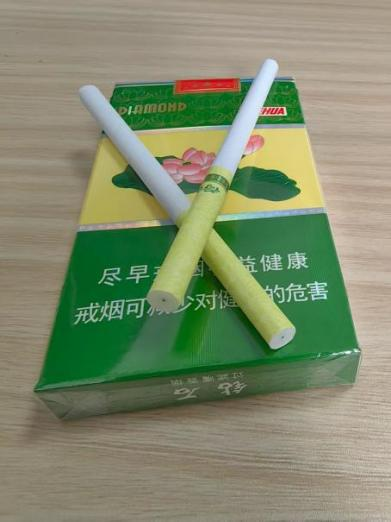


**Supplementary Figure 1.** Cigarette with yarn in the filter rod from China Tobacco Hebei Industrial Co., Ltd.


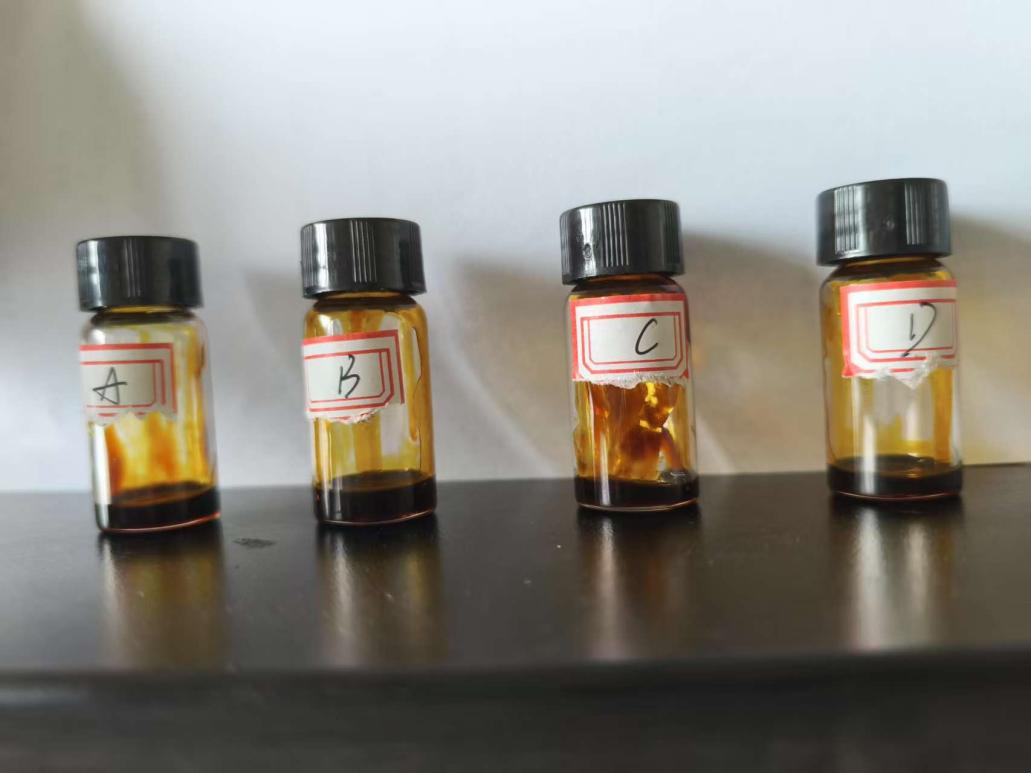


**Supplementary Figure 2.** crude extract of waste tobacco leaves from Longyan (A), Lanxiong (B), Zunyi (C), and Yongzhou (D)


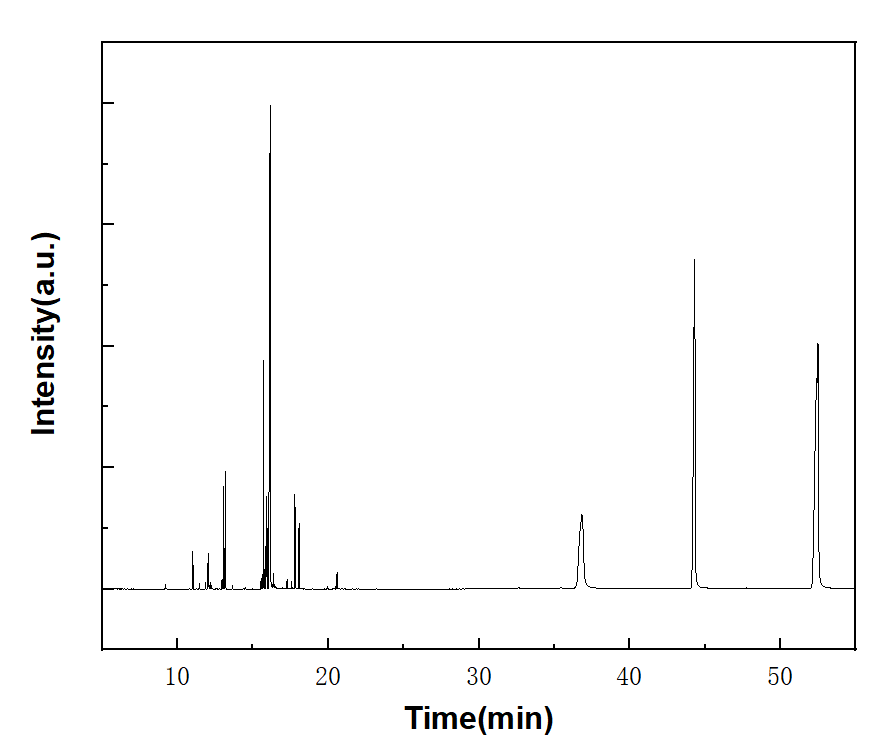


**Supplementary Figure 3.** Gas chromatography analysis of the peppermint essence


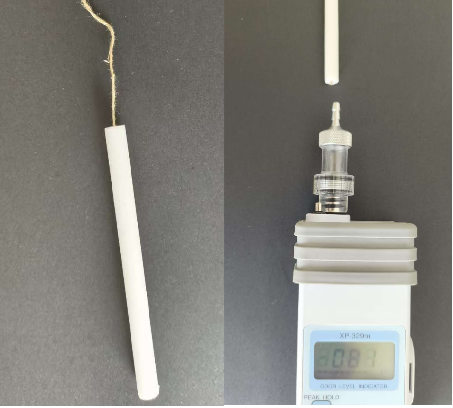


**Supplementary Figure 4.** Filter stick containing fragrant yarn and its fragrance value

**Supplementary Equation 1.** Kubelka-Munk equation

$\frac{K}{S}=\frac{(1-\rho_{\infty})^{2}}{2\times\rho_{\infty}}$ Eq. S1

**Supplementary Table 1. The color difference of the dyed yarns**

|  | 0.5wt% | 0.5wt% | 0.5wt% | 1wt% | 1wt% | 1wt% | 1.5wt% | 1.5wt% | 1.5wt% |
| --- | --- | --- | --- | --- | --- | --- | --- | --- | --- |
| A | 24.68 | 24.72 | 26.96 | 29.05 | 29.81 | 29.36 | 30.82 | 30.53 | 30.05 |
| B | 27.09 | 26.6 | 26.31 | 29.1 | 28.9 | 29.11 | 29.91 | 29.7 | 30.18 |
| C | 32.99 | 32.82 | 32.87 | 34.98 | 35.32 | 35.46 | 35.88 | 36.61 | 35.57 |
| D | 24.75 | 24.67 | 24.25 | 27.17 | 27.29 | 26.77 | 29.81 | 30.07 | 29.85 |
| The SPSSAU project (2025). SPSSAU. (Version 25.0) [Online Application Software]. Retrieved from https://www.spssau.com. | | | | | | | | | |
